# Views of Farmers and Industrial Entrepreneurs on the Iberian Pig Quality Standard: An In-Depth Interview Research Study

**DOI:** 10.3390/ani10101772

**Published:** 2020-09-30

**Authors:** Alberto Ortiz, Natalia Carrillo, Ahmed Elghannam, Miguel Escribano, Paula Gaspar

**Affiliations:** 1 Meat Quality Area, Center for Scientific and Technological Research of Extremadura (CICYTEX-La Orden), Junta de Extremadura, 06187 Guadajira, Badajoz, Spain; alberto.ortiz@juntaex.es; 2 Department of Animal Production and Food Science, School of Agricultural Engineering, University of Extremadura, Avda. Adolfo Suarez, s/n, 06007 Badajoz, Spain; ncarrillh@alumnos.unex.es (N.C.); mescriba@unex.es (M.E.); 3 Department of Economics, School of Agricultural Engineering, University of Extremadura, Avda. Adolfo Suarez, s/n, 06007 Badajoz, Spain; ahmedelghannam66@gmail.com

**Keywords:** Iberian pork, quality standard, qualitative analysis, in-depth interviews

## Abstract

**Simple Summary:**

This paper aims to assess the main opinions of farmers and industrial entrepreneurs on the implementation of the current Spanish Iberian Pig Quality Standards regulation as well as on the processing technologies of Iberian cured products. The study is based on a qualitative research process through in-depth interviews, and has allowed the identification of aspects that can be improved both at the level of the Iberian meat industry and in the administrative processes in the view of the main actors of the Iberian pork sector in Spain. The aspects of the Quality Standard related to the protection of the base of the Iberian breed, the conditions of production in the traditional system (the montanera), as well as the ripening time of the products were mostly supported by the farmers and industrial entrepreneurs. However, they showed certain inconformity with the requirements established by the Quality Standard for other production systems such as the non-free-range fodder-fed and free-range fodder-fed, therefore they demanded changes in these aspects.

**Abstract:**

Since 2014, the Quality Standard for Iberian meat, leg ham, shoulder ham and dry-cured loin has regulated production factors and processes involved in the raw material and manufactured products from Iberian pigs, the most important pig breed in both population size and economic importance of the southwest Iberian Peninsula. Regarding the changes to the Quality Standard that industrial entrepreneurs and farmers are currently demanding, a qualitative research study has been developed through 14 in-depth interviews with the purpose of understanding the perception of Iberian pig farmers and industrial entrepreneurs of the requirements of the currently-effective Quality Standard, as well as the conditions under which this is being applied. The results showed a consensus amongst the majority of the participants in aspects such as the maintenance of the breed base as 100% Iberian for reproductive females, weight and age requirements at the time of slaughter for the montanera category and the manufacturing lengths for dry-cured products. On the other hand, there were discrepancies between the requirements defined by the Quality Standard and those requested by the respondents for the non-free-range fodder-fed and free-range fodder-fed categories, with the industrial entrepreneurs and farmers being inclined towards the reduction in the age of slaughter of the former and the distinction in the production conditions of the latter.

## 1. Introduction

Pork meat consumption represents a major part of the diet of the European countries, with pork being the most consumed and preferred meat, before chicken and beef [1]. In recent years there has been an increasing demand of meat products deriving from autochthonous breeds that are reared in extensive systems, which is potentially due to a positive perception of society as to their contribution in the preservation of the environment [2], animal welfare [3], as well as the perceived high quality of the derivative products [4].

This is the case of the Iberian pork, the most important pig breed in both population size and economic importance of the southwest Iberian Peninsula [5]. High acceptance and demand of Iberian products have enabled the development of the industry involved, which still has major problems to deal with, such as the great variability of factors associated with the various stages of the production chain, giving rise to a diversity of production models and therefore differences in the final quality of the products. These factors include the genetic background of animal [6], the production system, the feed provided to the animal, especially during the final finishing stage [7]. Additionally, animal age and weight at the beginning of the finishing stage [8] and at the time of slaughter [9,10] are factors to consider in the quality of the products derived. Further, this variability to which the Iberian pork products are subjected makes it difficult for the detection of any fraudulent activity that may take place within the industry [11].

The first Spanish Iberian Quality Standard [12] emerged in 2001 within this context with the main purpose of guaranteeing and defining the quality traits and control process, regulating Iberian pig production factors and the commercialization of their derived dry-cured products. Its subsequent amendment in 2007 [13] extended its scope of application to pork cuts that are commercialized as fresh meat. The current Spanish Iberian Quality Standard—known as the Quality Standard for Iberian meat, leg ham, shoulder ham and seasoned pork loin—came into force in 2014 [14] (hereinafter, QS) as an attempt to clarify and provide transparency to the industry, as well as providing a simpler perception of the market products and their various agreed quality standards grouping them under a new labeling system. Thus, four commercial categories (labels) were defined: the Black label (100% Iberian breed acorn-fed pigs), Red label (at least 50% Iberian breed acorn-fed pigs), Green label (at least 50% Iberian breed pigs, reared in pastures (dehesas) and fed on fodder and grass) and White label (at least 50% Iberian breed pigs, reared in confinement and fed only on fodder (Table 1).

Thus, the commercialization of the products under the aforementioned labels requires an effort by farmers, industrial entrepreneurs and traceability and control systems. This in turn means an increase in the production costs, which at times is not compensated by the selling price, given that all this effort to improve the industry is not always perceived and translated in the consumer purchasing decisions [15].

Several years since the implementation of the current QS, there is certain degree of disagreement amongst the involved stakeholders, i.e., farmers and industrial entrepreneurs, with regards to some of the requirements set out in the QS for the various categories, specifically in relation to production factor in the farm, certification process and technological processing of the products. Amongst others, one of the factors raising most of the interest and controversy within the production aspects is the age of slaughter of animals, which is determined according to production system (montanera, free-range fodder-fed and non-free-range fodder-fed) and, disregarding the animal Iberian breed percentage. Although traditionally, a long production cycle has been the preferred option for the Iberian breed in order to obtain high-quality products [8], the improvement in the production parameters deriving from the use of the Duroc breed [6]—authorized by the current quality standard—could generate a misalignment between the QS’s required age of slaughter and the farmers’ interests, who broadly use this breed in order to increase productivity, reduce production cycles and therefore costs. Nevertheless, in spite of the relevance of the age of slaughter [16], as far as we are concerned, there are no studies that explore the recommended age in association to genetics, and there are only few studies that assess its influence on the quality of the meat derivatives [8], [17] and therefore that may combine the demands and interests of farmers and consumers alike. On the other hand, there is a clear lack of definition of the feeding and rearing system used for pigs under the Green label category (free-range fodder-fed animals), a fact that has even led to this category being excluded from sensorial studies on account of the lack of uniformity of its production aspects [18].

Additionally, the current QS does not contemplate any measures to help overcome the seasonality to which Iberian products are subjected—especially montanera Iberian dry-cured loins, which are launched to the market in the summer months with lesser demand, and considering that the period of greatest consumption of this type of products is from November to December (because of Christmas season), there is a gap between industry and consumer demand. In order to overcome this situation, industrial entrepreneurs use practices such as freezing the raw material prior to its technological process of curing, but the lack of European Regulations for the freezing of animal products [19], together with scarce scientific literature [20,21], has led to a situation of uncertainty regarding how this practice may affect the quality of the end product, which in turn has made it difficult to regulate and control it at the industrial level.

Given the above concerns and the over four years since the implementation of the QS, it is vital to assess the current QS, as well as any improvement proposals made by the stakeholders, and thus contribute to form a bases for a decision tool that may focus on these specific industry and administrative issues.

In this context our purpose was to understand the main views of farmers and industrial entrepreneurs on the application of the current QS for the Iberian product through an in-depth interview qualitative research study, in order to identify the limitations and opportunities for the commercialization of Iberian products in the current environment.

## 2. Materials and Methods

**Figure 1:**
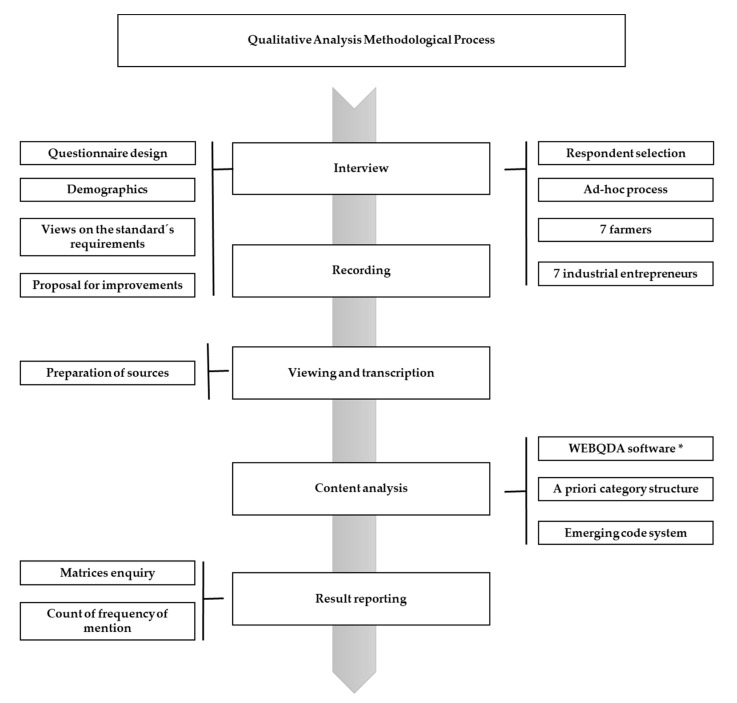
Methodological process followed in order to carry out the in-depth interviews. * WebQDA Software V 3.1. is a qualitative data software analysis. It allows editing, viewing, linking and organizing different sources as text documents or audio files. It can create categories, codes, manage, filter, search and question the data in order to answer the questions that emerge in the research. WebQDA Software has been developed by the partnership Universidade de Aveiro; CIDTFF; Esfera Crítica and Ludomedia (https://www.webqda.net/).

This research study has been based on a qualitative method throughout in-depth interviews on account of its exploratory nature and because of the high level of controversy amongst the various stakeholders involved in this industry. The research team selected a widely-recognized semi-structured model that is largely used in these types of research studies [22]. Two versions of the interview script were designed and adapted to the purposes of the study, one per type of stakeholder (farmers and industrial entrepreneurs). Figure 1 presents the full methodological process followed for the research study.

### 2.1. Data Collection

For the purposes of this research study, all the interviews were face to face interviews carried out at the workplace of the respondents. The final sample was selected following a convenience sampling process, which is a non-probability type of sampling that is broadly used in qualitative research [23,24]. For this piece of research, the respondent selection process was progressive, as interviews were conducted at the same time as the respondents were classified by characteristics. Subsequently, from the information obtained, new farmers and industrial entrepreneurs with different characteristics from the ones that had already been interviewed were sought. This particular selection process was adopted with the purpose of covering the various types of businesses in terms of size (small, medium and large), categories (or labels) of products sold (Black, Red, Green and White label) and production type; both in the case of farmers (closed cycle, only montanera/fodder, other integrations) and industrial entrepreneurs (dry-cured products, fresh products, both) and finally, the various market plans for the products (local, national or international).

Subsequently, a telephone conversation was held with the selected respondents, where the purpose of the research study was explained, and they were asked cooperation in the conduction of the semi-structured interviews. Additionally, they were assured that the data provided would be confidential in compliance with the Spanish Data Protection Act. Fourteen in-depth interviews were conducted in total, seven of which were conducted with farmers and the other seven with industrial entrepreneurs. This number of interviews was in line with that of other qualitative research studies carried out using semi-structured interviews [25]. All the participants were asked for their consent to be audio recorded during the interviews and they all accepted. The average length of each interview was approximately 120 min.

### 2.2. Interview Script Design

The interviews were structured in five sections designed to meet the purposes of the research study. As Table 2 shows, the interviews included similar questions for farmers and industrial entrepreneurs, although in some cases there were specific questions for each group.

### 2.3. Data Analysis

Once the interviews had been conducted, the recordings were transcribed and the information collected was analyzed by means of a Content Analysis methodology [26]. Content analysis is method attempting to obtain valid and applicable inferences from the texts with the purpose of reducing the original material [27]. The webQDA software (v. 3.1, Ludomedia, Oliveira de Azeméis, Portugal) was used for the purpose of this analysis, establishing a tree structure with a priori categories and an “emerging” code system linked to each category. Building a priori categories consists of establishing a preliminary hierarchical system prior to reading the documents, which for the purposes of this research study was predetermined by the items included in the semi-structured interview. Coding is a systematic way of developing and refining the data interpretation. The coding process includes the collection and analysis of all the data relating to subjects, ideas, concepts, interpretations and propositions [28]. Specifically, emerging coding is characterized by being inductive or open with codes being generated as the information is processed, in a way that as the data are being read or interpreted, new amendments emerge. Subsequently, the frequency of mention of each opinion was obtained from the views of each respondent that were coherent with the ideas or concepts contained in each code with respect to the total of responses, converted to a percentage [29].

## 3. Results

The results are presented according to the a priori categories established in the methodological process. These are a total of eight categories of which the first four are categories related to the requirements of the QS at the farms, the next two have to do with the administrative aspects that the QS also establishes, such as certification and inspection processes as well as product labeling. The last two categories refer to aspects related to the processing of the products by the manufacturers.

### 3.1. Farmers and Industrial Entrepreneur Views on the Requirements of the Quality Standard for Farms

#### 3.1.1. Requirements in Terms of the Breed Base of the Reproductive Animals

**Figure 2:**
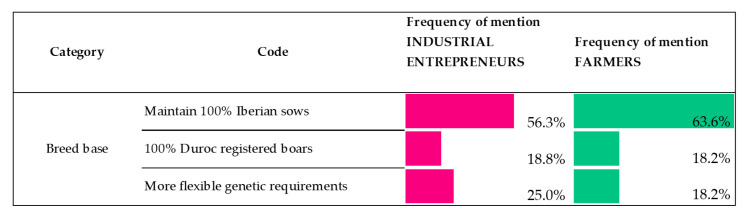
Industrial entrepreneur/farmer views on the requirements of the QS in terms of breed base. (Scale: frequency of mention of each opinion. Percentage is based on responses in the given code out of all responses to equal 100%).

With the purpose of protecting the genetic value of the Iberian breed, the QS establishes that all females must be 100% Iberian breed. These can be used for the Iberian female x Duroc male cross-breeding, whereas the Duroc breed is reserved for the male line, provided always that both are registered in the herd book. In Figure 2 we can see how in their majority, both farmers and industrial entrepreneurs who are asked about this, are inclined towards the protection and preservation of a 100% Iberian female, with 63.6% and 56.3% in frequency of mention, respectively.

The following were some of the literal comments made by the respondents:

“This has been one of the major contributions of the QS to the industry because it has maintained production at the same time as preserving the pure Iberian female”.

“It is great for the Standard to protect the Iberian female because, otherwise, this would all get out of hand”.

Another idea that was contributed during the interviews by industrial entrepreneurs and farmers was the need to guarantee the breed purity of Duroc boars in the farms where they decide to have cross-bred animal products, with over 18% in the frequency of mention both by farmers and industrial entrepreneurs.

Lastly, some views were in favor of making the genetic requirements more flexible (Figure 2), mainly amongst the industrial entrepreneurs (25.0% of frequency of mention), in a way that the QS would allow animals whose characteristics were compatible with the breed standards, even when they were not registered in the herd books. This goes against the currently effective regulations which are supported by Iberian Breed Association [14].

#### 3.1.2. Requirements in Terms of Minimum-Weight Gain at the Finishing Stage and Minimum Carcass Weight

According to the current QS, the average weight of the animal lot at the beginning of the montanera stage (Black and Red labels) must be between 92 and 115 kg, gaining a minimum of 46 Kg during at least 60 days. In the case of animals produced under the free-range fodder-fed and non-free-range fodder-fed categories (Green and White labels, respectively), the QS does not establish the weight gain applicable during the finishing stage. As a common feature for all categories, the carcass weight must be greater than 115 kg in cross-breeds (Iberian x Duroc), and 108 kg in pure Iberian animals.

**Figure 3:**
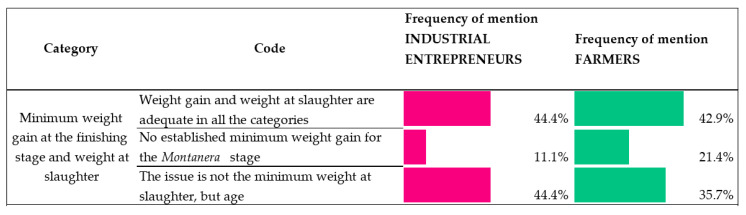
Industrial entrepreneur/farmer views on the requirements of the QS (Quality Standard) in terms of weight gain at the finishing stage and minimum weight at slaughter. (Scale: frequency of mention of each opinion. Percentage is based on responses in the given code out of all responses to equal 100%).

Almost half of respondents when asked for this requirement, both from the farm and the industrial backgrounds, were of the opinion that the weight gain and weight at slaughter were adequate (in order to achieve the minimum carcass weight) in all categories (42.9% and 44.4% in frequency of mention, respectively) (Figure 3). Examples of comments in this line are:

“With regards to weight, I think it is important to maintain the limits established by the QS, because a pig that does not reach the adequate weight will not prove an adequate carcass later on”.

Nevertheless, some interviewed farmers would agree to not establishing minimum-weight gains for animals reared under the montanera system (Black and Red labels) (21.4% of frequency of mention).

On the other hand, a large proportion of farmers, and especially industrial entrepreneurs, (35.7% and 44.4%, respectively) pointed out that the issue might not be so much in the minimum carcass weights required by the QS (108 to 115 kg) but in the minimum age of slaughter, which was inferred from the interviews through comments such as the following:

“I think it is OK, although the industry complains about it being a bit high and with a little less weight they would have better selling hams, especially because the new Duroc hams have caused the ham meat yield to increase and, back then, when the Standard was published in 2014, it was assumed that a 115 kg carcass would yield 7–8 kg hams, which are easy to sell. They are now finding this is not the case, the average weight per ham is 8.5 to 8.6 kg which is way higher than the expected average”.

#### 3.1.3. Requirements in Terms of Feeding at the Finishing Stage

**Figure 4:**
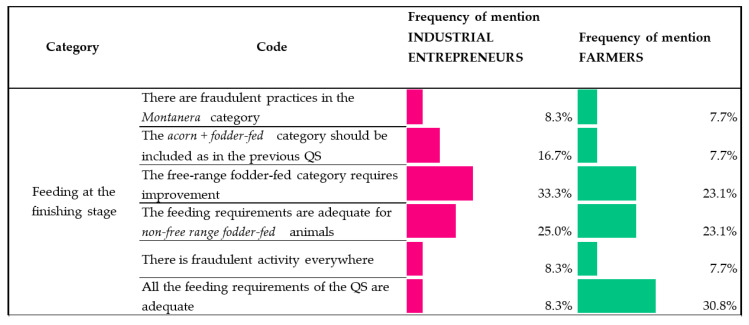
Industrial entrepreneur/farmer views on the QS (Quality Standard) feeding requirements. (Scale: frequency of mention of each opinion. Percentage is based on responses in the given code out of all responses to equal 100%).

The QS establishes that pigs reared under the montanera system (Black and Red labels) must be only fed on natural resources (acorns and grass); free-range fodder-fed pigs (Green label) must feed on fodder made of cereal and legumes, without prejudice to the use they may make of the natural resources, and that non-free-range fodder-fed animals (White label) are fed only on fodder. Figure 4 collects the respondents’ main views on the aspects set out by the QS in terms of the feeding at the finishing stage.

The perception that “there are fraudulent practices with animals reared in the montanera production system” represented 8.3% and 7.7%, in frequency of mention by industrial entrepreneurs and farmers, respectively. On the other hand, there were some which were in favor of “including again the free-range acorn + fodder-fed animal category that was contemplated in the previous QS” (16.7% and 7.7% of frequency of mention by industrial entrepreneurs and farmers, respectively).

With regards to the animal feeding requirements for free-range fodder-fed animals (Green label), most of the views of the industrial entrepreneurs and farmers, with 33.3% and 23.1% in frequency of mention, respectively, concluded that it was necessary to improve the current QS requirements.

Participants generally showed a consensus with the requirements of the QS in terms of feeding in the non-free-range fodder-fed category (White label).

Lastly, in spite of not being a majoritarian view, some participants expressed the idea that there are generally fraudulent practices in the industry in terms of the feeding at the finishing stage (coded as “There is fraudulent activity everywhere”; Figure 4), which attempts to classify animals fed on fodder as montanera animals.

#### 3.1.4. Requirements in Terms of the Minimum Age for Slaughter

For each of the production systems set out in the QS, a series of requirements is established for minimum age of slaughter: for pigs reared under the montanera system (Black and Red labels), the minimum age for slaughter is 14 months and for free-range fodder-fed (Green label) and non-free-range fodder-fed (White label) the age is 12 and 10 months, respectively. 

**Figure 5:**
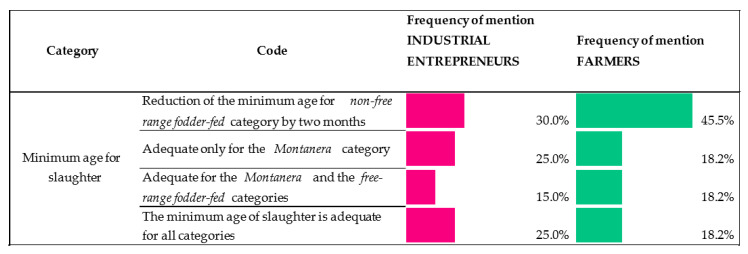
Industrial entrepreneur/farmer views on the QS (Quality Standard) requirements for age of slaughter (Scale: frequency of mention of each opinion. Percentage is based on responses in the given code out of all responses to equal 100%).

A percentage of industrial entrepreneurs and farmers (30.0% and 45.5% in frequency of mention, respectively) were supportive of a reduction in the age of slaughter by two months for non-free-range fodder-fed pigs (White label), going from 10 to 8 months (Figure 5). Some examples of these comments were:

“The fair thing to do would be to adjust the age of the animal in order to obtain a product that is more adapted to the market and able to make it stable”.

“Thanks to the existing technological advances, pigs cannot be slaughtered at the age of 10 months, which would not comply with the requirements of the market; this is where by trying to improve the pig or other parameters, we may forget that the purpose is to sell it in a market demanding an 8-month-old pig, not because it is 8 months old, but because this is the age at which it can be sold into the market; the consumer does not want a ham that weights more than 7 kg”.

“The age of slaughter should be earlier for non-free-range fodder-fed animals at least by two months”.

“The genetics of pigs have been improved and currently 8-month-old pigs or younger would be ready for slaughter”.

In the case of animals that are fed under the montanera system, industry entrepreneurs and farmers thought—with 25.0% and 18.2% of frequency of mention, respectively—that the currently-established age of slaughter is adequate. Amongst their statements, the following can be highlighted:

“Animals like those my father used to rear, montanera all his life. If I tell him that the current minimum is 14 months old, he will laugh at me, because that is so little time, if you think of the time an animal with good qualities and traits needs to fully rear”.

Finally, other farmers and industrial entrepreneurs also expressed their views on keeping the age of slaughter proposed by the QS for the various production systems as they thought they were adequate (montanera, free-range fodder-fed and non-free-range fodder-fed), with 18.2% and 25% in frequency of mention, respectively.

### 3.2. Farmer and Industrial Entrepreneur Views on the Certification Processes and the Product Labeling

#### 3.2.1. Views on the Certification/Inspection Process

**Figure 6:**
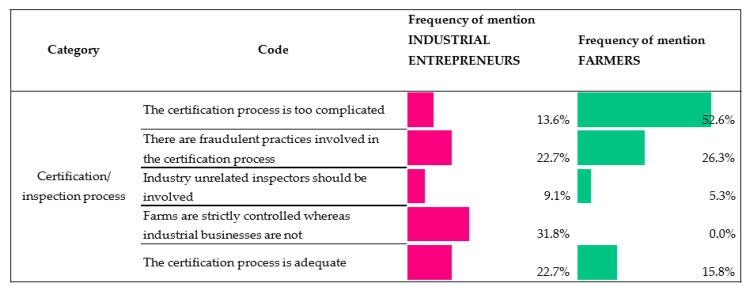
Industrial entrepreneur/farmer views on the requirements of the QS (Quality Standard) in terms of certification/inspection. (Scale: frequency of mention of each opinion. Percentage is based on responses in the given code out of all responses to equal 100%).

The certification and inspection processes are key for Iberian pigs and their meat products, as they are the tool that guarantees they comply with the QS both at the production stage in the fields and the manufacturing and commercialization stages. In this regard, various matters were dealt with during the interviews, with the results being shown in Figure 6.

Notably, the participating farmers (52.6% in frequency of mention) complained about the excessive control measures they are required to submit their practices, especially at production level, with the purpose of ensuring the quality of the inspections, as well as the amount of documents that they need to provide in order to prove their situation.

#### 3.2.2. Views on the Labeling Process

In terms of the labeling of the Iberian pork products, the majority of the industrial entrepreneurs mentioned the excessive importance that the QS places on the font size and type to be used, which was considered irrelevant by the above in contrast with more important aspects, such as the information that is provided to the consumer.

The participants thought it was much more important to mention the animal’s breed purity (whether it is pure or a cross-breed with Duroc), the production system (extensive or in confinement) and even indicating the age at which it was slaughtered.

On the other hand, they also felt that it would be important to select the key words to be used on each label depending on the type of animal (words such as Iberian, black leg, free-range fodder-fed, etc.).

### 3.3. Industrial Entrepreneur Views on the Processing of the Products by the Manufacturers

#### 3.3.1. Views on the Freezing Process of Raw Materials

Part of industrial entrepreneurs saw freezing as a feasible solution to manage the excess product in the industry and to adapt it to market demand, with 19.2% mentions. On the other hand, with the same frequency of mention, industrial entrepreneurs pointed that freezing was a solution for importing and exporting products since the useful life of fresh meat is quite limited. However, the participants pointed out that consideration must be given to the alterations the product might suffer in terms of quality. Thus, the same proportion of respondents indicated that freezing affects the quality of the products as those stating that it does not (15.4%).

**Figure 7:**
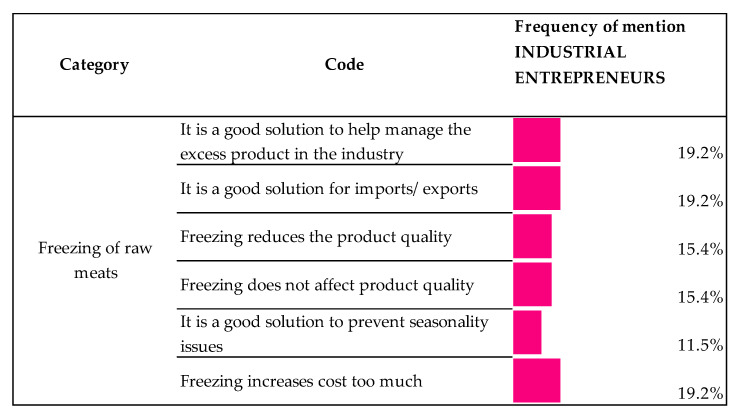
Industrial entrepreneur views on the requirements of the QS (Quality Standard) in terms of product freezing. (Scale: frequency of mention of each opinion. Percentage is based on responses in the given code out of all responses to equal 100%).

On the other hand, the seasonality to which the Iberian products are subject, especially those deriving from pigs reared under the montanera system (Black and Red label), makes it necessary for this industry to innovate in order to adapt production to consumer demand. A potential solution would be freezing the hams raw material prior to their curing process as mentioned by the industrial entrepreneurs in this research study with 11.5% frequency of mention (Figure 7).

On the other hand, other opinions were related with the idea that freezing the product increases the costs.

Some contributed the above opinions with statements such as:

“The freezing process clearly increases the cost of the product, which does necessarily translate into an increase in the final product”.

“Freezing the hams is a solution that would help optimize the facilities of this industry”.

“Slaughtering and acorn-fed animals have a seasonal component to them, and Christmas is when most of the acorn-fed product is sold. For example, loin is always frozen in preparation for the Christmas demand”.

#### 3.3.2. Views on the Minimum Obligatory Maturity Time

**Figure 8:**
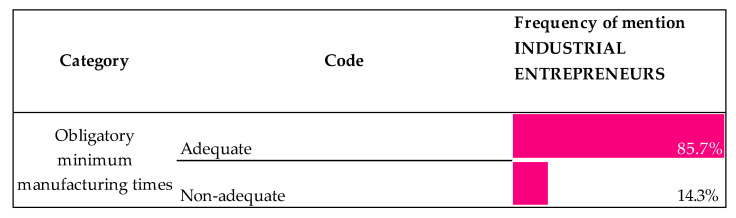
Industrial entrepreneurs’ views on the requirements of the QS (Quality Standard) in terms of the maturity times. (Scale: frequency of mention of each opinion. Percentage is based on responses in the given code out of all responses to equal 100%).

An aspect that is defined by the standard is the minimum manufacturing times, which range from 600 to 730 days for leg hams, 365 days for shoulder hams and 70 for dry-cured loins. It is important to point out that these timings are minimums, and the final maturity period can be much longer, depending on the manufacturing method used in the industry. In this regard, Figure 8 portrays the views of respondents on these maturity times.

As Figure 8 shows, with over 85% in frequency of mention, industrial entrepreneurs stated that the maturity times for Iberian products—leg hams, shoulder hams and dry-cured loins—were adequate.

“The acorn-fed product fully complies with it under the traditional system and in the case of the free-range fodder-fed product, it must comply too, as it is the case of a greased animal. If anyone were able to put it out earlier in the market by artificial means, they will do it, but… to what extent should we tell them: put it out now! One thing is clear: quality takes time and the standard is the reference stating the times in order to prevent us from going the fastest way possible”.

## 4. Discussion

The regulation of the breed base of reproductive animals was certainly the main motivating aspect for the implementation of the first QS in 2001 [12] and this has been maintained through to the current QS [14]. Both sectors, farmers and industrial entrepreneurs, were of the opinion that this requirement has given rise to a positive increase in the number of Iberian animals [30], thus preventing the loss of purity of the Iberian breed against the cross with Duroc, as was the case before the application of the first Iberian QS [11,31]. On the other hand, the main reasons for the need to guarantee the breed purity of Duroc boars may be associated with characteristics of the Duroc breed itself, such as an improvement in the production parameters, prolificacy, higher energy efficiency, lean meat yield and growth ratio of the cross-bred animals, which are consistent with the scientific literature [6]. In spite of this requirement being effective since the QS was published in 2001, it became more demanding with the application of a new inspection protocol in 2017 [32]. This gave rise to an increase in the demand of registered pure Duroc boars and a price increase [33].

With respect to the minimum-weight gain at the finishing stage and minimum carcass weight, the fact that some responders, mainly farmers, considered not to establish a minimum-weight gain during montanera could be explained due to years of bad weather resulting in lack of natural resources (acorns and grass), and therefore inability to reach the minimum weight gain threshold required—46 kg— on only natural resources. On the other hand, the large proportion of respondents that indicated that the minimum age of slaughter was the main issue to solve, instead of the minimum-weight gain during the finishing stage, could be attributed to financial terms. Thus, most of the animals currently being slaughtered in Spain are those yielding White and Red label products, being 80% and 14%, respectively, of the total slaughtered animals [34], which are therefore Iberian-Duroc crossed animals. Since the QS establishes the age at slaughter for each category, regardless of the breed purity, the greater growth speed of Iberian × Duroc crossed animals [6] could lead to animals reaching the age of slaughter required by the QS with heavier weights to those the farmer deems optimum to render the most profit [35]. On the other hand, industrial entrepreneurs are faced with the difficulty of processing hams from animals which are overweight, especially leg hams above 8 Kg, which forces the increase of curing and stocking times, as well as the commercializing of products that are not in line with the current trends and habits of consumption [36].

Regarding to the requirements in terms of feeding at the finishing stage it should be highlighted that respondents think it is not well defined for free-range fodder-fed animals (Green label). This may be due to the lack of specific criteria to differentiate the degree of intensiveness in which the animals are reared (animals per hectare), as well as the percentage of feed the animal has eaten from natural resources in the dehesa and from fodder. This situation gives rise to a lack of an adequate distinction within the free-range fodder-fed category that is translatable into a differentiated type and therefore into the price of the derived products, thus acting in detriment of the profitability threshold of the extensive producer. In this sense, the scientific literature we find on the relevance of the production factors [15] or the influence of the various QS Iberian packaged products [18] on consumer preference have omitted the Green label as a commercial category due to the lack of uniformity of the farming conditions and therefore the high degree of heterogeneity of the end product.

With respect to the requirements in terms of the minimum age for slaughter, the relative high portion of respondents who agreed with the adequacy of the slaughtering age established by the QS for montanera animals could be explained by the greater presence of 100% Iberian animals in the montanera production system (Black label), which, according to the existing scientific literature, grow and mature slowly and have low meat yield [16], which indicates the requirement for them to have a longer production cycle in order to obtain quality products. In this line, there are studies which analyzed the traits of the carcass and the meat quality of pure Iberian pigs by age, concluding that the animals being introduced to the montanera rearing system at a younger age might not be mature enough to make the most of it [17], and could led to poorer growth rate, as well as carcass quality and fat traits [8]. As far as we are aware, there are no studies on the impact of the age of slaughter on cross-breed pigs reared under the montanera system (Red label). The free-range fodder-fed animal (Green label), however, has a more complex context, due to the great variability of the production factors. On the one hand, extensive farmers state these animals are very similar to the pigs reared under the montanera system and therefore, the age at slaughter is adequate. On the other hand, intensive farmers state that the age of slaughter should be reduced or maintained due to its similarities with the non-free-range fodder-fed animals (White label). Contrarily, most of the industrial entrepreneurs and farmers agreed with a reduction in the age of slaughter by two months for non-free-range fodder-fed pigs (White label), maybe because animals reared under this category come from Iberian × Duroc crossed animals and therefore, they grow faster and yield more meat [6] in comparison to the pure Iberian breed, and they could reach the optimum weight for slaughter at a younger age.

Moving to certification processes and the product labeling, the high frequency of mentions reporting the too complicated certification process by farmers could be explained by overlap between the various institutions carrying out inspections and the certifying companies in the first place, and then the Autonomous Communities Authorities and Interprofessional Iberian Pig Association (ASICI, in its Spanish abbreviation), which leads to a general sense of irritation in the industry [37]. To overcome this situation, farmers and industrial entrepreneurs suggested that inspections could be conducted by independent experts unrelated to the farming industry, but with sufficient knowledge and skills to perform the job, since the main purpose of this is to prevent industry fraud. 

On the other hand, the importance given by respondents to mention the animal’s breed purity in product’s labeling may be due to the fact that participants understood that breed is a quality indicator for the consumer, in spite of the fact that various studies may not support the same position, demonstrating that consumer places much more importance on the type of feed than the breed [38] and concluding, additionally, that consumers cannot distinguish—from the sensorial point of view—between dry-cured products coming from pure Iberian pigs or crossed with Duroc, when the feed is the same [15].

In regard to the views on the freezing process of raw materials, there was no consensus about its impact on quality. In this line, no detriment in quality has been reported in Iberian pork meat after a year and a half frozen [39]. On the other hand, freezing raw material from pigs under montanera system could help to overcome the seasonality to which these products are subjected. However, this practice is not contemplated by the current QS [14] nor by the European standards dealing with freezing animal meats [19]. As far as we are concerned, there are few research studies relating to the freezing of meats prior to their curing process and mostly carried out on leg ham from commercial pigs [40,41]. Few studies deal with such topic in leg hams from Iberian pigs [42,43,44], and that analyze its effects on Iberian loins [21], so further studies being required in order to assess the effects of freezing on the final product as well as consumer acceptance.

Lastly, where a consensus was observed was in the fact that freezing the product increases the costs. In the first place, because a frozen product translates into money that is not circulating and, in the second place, because the maintenance of the freezing process implies a relevant cost for any industry.

With respect to the views on the minimum obligatory maturity time, the general agreement about the manufacturing length follow the lines of the scientific literature. Research studies concluded [45] that consumers prefer leg hams that have a long process to mature, as they positively associate this fact with an improvement in texture, flavor and aroma. We can conclude that maturity time is a parameter that does not give rise to much dispute, as the nature itself of the production process defines minimum times that must be observed.

## 5. Conclusions

A qualitative research study involving the use of in-depth interviews allowed the stakeholders to identify key aspects for future potential modifications in the current Iberian QS. Our findings showed industrial entrepreneurs and farmers were of the same opinion in aspects of the QS that have a significant impact on the profitability, the production yield and the quality of the end product. There was general consensus in terms of the preservation of the Iberian breed for sows, the elimination of the minimum weigh gains in animals under the montanera system as well as establishing an additional difference within the free-range fodder-fed category. Additionally, the participants shared the view that the age of slaughter established by the QS for non-free-range-fodder-fed animals is too high, which leads to a detriment in the commercial value of derivatives due to excessive weight.

On the other hand, this research study highlighted the dissatisfaction of the participants with the excessive bureaucracy required for the commercialization of products under the current QS. This aspect could potentially pose a risk for the industry, as farmers may be inclined to abandon their activities given the highly atomized environment with a lack of qualified personnel that characterizes the Iberian sector.

With regards to technological processing, the participants thought freezing was an adequate solution in order to manage the balance between production and demand, which was particularly relevant for the animals reared under the montanera system. They believe that any future amendments to the QS should take the regulation of such practice into account.

## Figures and Tables

**Table 1:** Requirements for the production aspects of the various categories of Iberian products and manufacture minimum times according to the current Quality Standard (QS).

**Production Aspects**	**Commercial Label**			
	**Black Label** 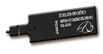	**Red Label** 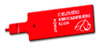	**Green Label** 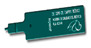	**White Label** 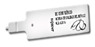
Breed (100%, 75% and 50% Iberian) provided that the female is 100% Iberian breed and the male is Duroc breed, both registered in a genealogic tree	100%	75%, 50%	100%, 75%, 50%	100%, 75%, 50%
Management system (the animals can be reared under various levels of intensiveness)	Extensive		Semi-intensive	Intensive
	(0.25–1.25 animals/ha) subject to the wooded area available and the availability of acorns		At least 100 square metres/animal when the live weight exceeds 110 kg	At least 2 square metres/animal when the live weight exceeds 110 kg
Weight and minimum weight gain during the finishing stage	46 kg for over 60 days		At least 60 days prior to slaughter	
Minimum age at slaughter	14 months		12 months	10 months
Feed allowed during the finishing stage for each category	Feed based only on acorn, grass and other natural resources found during *Montanera* in the *dehesa*		Feed based on fodder made of cereal and legumes with the possibility for the animals to either fully or partially rearing in *Montanera*	Fodder made of cereal and legumes
Carcass minimum weight	115 kg, except for 100% Iberian animals, which will be 108 kg			
**Product**	**Leg Ham** 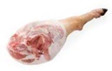		**Shoulder Ham** 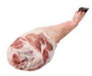	**Loin** 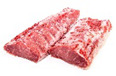
Minimum time of manufacture of the products	W < 7 Kg 600 days, W ≥ 7 Kg 730 days		365 days (regardless of weight)	70 days (regardless of weight)

W: weight; Own source based on the current QS [14].

**Table 2:** Script of the interviews conducted with farmers and industrial entrepreneurs.

Interview Design	Farmers	Industrial Entrepreneurs
Demographics	Information on the age, sex, education level, job title and experience in the position	
Type of farm/industry	Business activity (farming, industrial or both), number of employees and their distribution in the various departments, production type under the QS, commercialisation channels and sales	
	Nº of pigs sold per category	Main product type sold (fresh, cured)
	Animal breed base	Brands under which the products are commercialised
		Countries for export, if any
Views on the various aspects of the Quality Standard (RD 4/2014)	Views on the requirements of the QS in terms of breed base, feeding type, weight gain at the finishing stage and weight/age at time of slaughter	
	Views on the certification process for farms and industrial businesses	
		Manufacturing time for products according to the QS
Views on production seasonality and Iberian product demand	-	Strategies used to correct discontinuity of demand of Iberian products
	-	Freezing of the fine cuts prior to the curing process. Impact on the final quality of the product and production costs. Need to specify such practice on the label
Proposals for improvement	Applicable measures aimed at improving the identified deficiencies or others not referred to previously. Individuals responsible for their implementation.	

QS: Quality Standard.
